# Knowledge, Attitude and Practice of Community Pharmacists Toward Non-pharmaceutical Products in Saudi Arabia

**DOI:** 10.3389/fpubh.2022.771308

**Published:** 2022-04-29

**Authors:** Dalia Almaghaslah

**Affiliations:** Department of Clinical Pharmacy, College of Pharmacy, King Khalid University, Abha, Saudi Arabia

**Keywords:** community pharmacy, non-pharmaceutical products, Saudi Arabia (KSA), KAP (knowledge, attitude and practice)

## Abstract

**Introduction:**

Community pharmacy is a rapidly changing sector in Saudi Arabia. Customers visit local community pharmacies for a variety of reasons including disease-related advice, buying OTC medication, cosmetic products and re-filling a prescription for a chronic illness. The current study was conducted to fill the gap in the literature regarding community pharmacists' knowledge, attitude and practice toward non-pharmaceutical products.

**Methods:**

The study used a cross-sectional design. A total of 211 community pharmacists working in the Asir region, Saudi Arabia were included. The questionnaire was adopted from a previous study with the same purpose.

**Results:**

Products sold in community pharmacies were mainly pharmaceutical products (69.7%) compared to 30.3% non-pharmaceutical products. The most commonly sold non-pharmaceutical products were mother and baby products (26%) and skin care products (19%). Pharmacists showed good mean knowledge (3.96 out of 5), mean positive attitude (3.79 out of 5) and mean positive practice (3.32 out of 5).

**Conclusion:**

Community pharmacists showed considerable knowledge pertaining to non-pharmaceutical products. Respondents demonstrated positive attitudes toward the non-pharmaceutical products and were interested in expanding their knowledge on the topic through continuing education. Additionally, they showed social accountability by assuming responsibility for providing patient counseling on non-pharmaceutical products.

## Introduction

Community pharmacy is a rapidly growing sector in Saudi Arabia. It is the largest employment pharmacy sector in the country, and has recently been given more attention from the government (1). The government focused on public-private partnerships for effective delivery of preventative primary health care in Vision 2030 (2). The vision focused primarily on providing equitable accesses to healthcare for Saudi and non-Saudi residents of the country. This necessitates enhancing the value of health services through increasing the quality of services delivered through preventative and primary healthcare, while maintaining therapeutic care (3). The vision fulfilled the implementation of the suggested health transition programs: the corporatization of a health care model, a health insurance program and the purchase of health services, a private sector participation program, governance, e-health, and the workforce (3). The vision also aimed at increasing employment opportunities for Saudi nationals as well as increasing the number of female workforce in the job market (4).

The Ministry of Health (MOH) is the authority that regulates the community pharmacy sector in Saudi Arabia. Community pharmacies are usually managed by one to two pharmacists or by a pharmacist and an assistant. The work shifts are between 8 and 12 h a day, 6 days a week (5). Working in community pharmacy requires pharmacists to be licensed; registration for pharmacists at Saudi Commission for Health Specialties (SCFHS) involves passing the Saudi pharmacists licensure examination (SPLE) (3). Hence, pharmacists who joined the workforce must have had the minimum set of skills and competency to practice in the profession. Maintaining registration as a pharmacist requires obtaining Continuing Medical Education (CME) h. The CME requirement is 20 h per year minimum for a pharmacist's re-registration. These CME hours are divided into two categories; the first requires a maximum of 25 h of attending conferences, seminars, workshops, and training courses, as well as writing books, publishing scientific papers and conducting research (3). The second category, which requires a maximum of 15 h, includes internal activities, approved internet activities, panel discussions, and general workshops (3).

Community pharmacies in Saudi Arabia, like other countries, sell pharmaceutical products, i.e., prescribed medications, over-the counter (OTC) products and non-pharmaceutical products. Non-pharmaceutical products include but are not limited to herbal products, e.g. herbal teas, cosmetics, baby foods and formulas, mother and baby care products, food supplements, and sport foods such as protein powders and protein bars (2, 6). All pharmaceutical products including OTC products, prescribed medications, herbal products and food supplements are regulated by The Saudi Food and Drug Administration (SFDA) (5).

Community-based patient care services varied widely between countries. Previous studies conducted on Saudi adults revealed that consumers usually visit their local community pharmacies for a number of purposes including disease-related advice, buying OTC medication, cosmetic products and re-filling prescriptions for chronic illnesses (2). Recent transformation of healthcare system in Saudi Arabia resulted in expanding the role of community pharmacists to include more clinical services such as vaccination administration, training patients on the use medical devices, and preforming medication therapy management (7). Community pharmacists played an important role during COVID-19 pandemic by offering medication delivery services as well as providing remote patient counseling (8). On the other hand, patient care services provided in community pharmacies in the US include refills of emergency medication, renewals/extensions of prescribed medication, changes to doses or formulations, therapeutic alternatives, prescribing for minor ailments, the initiation of prescription drug treatment, ordering and interpreting laboratory investigations, and administering drugs by injection (9).

Effective patient counseling is a multifactorial task as it is influenced by the patient's expectations of their pharmacy visits and perspectives of the professional role of the pharmacists working in a community setting, pharmacist questioning skills, patient willingness to answer questions, and differences in pharmacists' and patients' perceptions of the diseases and the treatment (10). However, it has been reported that despite the efforts made by MOH and SFDA to ensure that community pharmacists follow the best practice in performing day-to-day tasks, i.e., drug dispensing and counseling, the services provided are still suboptimal (11). For example, there is unrestricted access to medications, as some prescription only medications can be obtained without prescription. Another example is dispensing medications without providing proper counseling which might lead to undesirable harm to patients (11, 12). A review article on community pharmacy services in developing countries including Saudi Arabia concluded that community pharmacy is commercial and profit-oriented (13). This business orientation in this area of practice, resulted in OTC medication being sold at higher rates. Drug representative persuasive endeavors and promotional activities offered to community pharmacists such as presents, sponsored meetings and advertising may affect the attitudes toward the drug company and its medical products (14).

The role of the pharmacist in the community setting is providing services and comprehensive patient counseling that result in the anticipated desirable outcomes of the medications and prevent unwanted adverse events (15). Community pharmacists are also expected to increase patient compliance with treatment, provide information regarding dosage regimens, and discuss any possible side effects of both prescribed and OTC medications.

Several local studies have assessed the performance and attitudes of community pharmacists toward prescription medications and OTC medication, as well as their involvement in providing public health services and pharmaceutical care, but none have evaluated their knowledge, attitude and practice toward non-pharmaceutical products sold in community pharmacies. Hence, this study was conducted to investigate community-based pharmacists *knowledge and training, attitudes and practice* toward non-pharmaceutical products. This study is the first to investigate community pharmacists' attitudes toward nonpharmaceutical products not only in Asir region but in Saudi Arabia.

## Methods

### Study Design

A cross-sectional self-administered questionnaire was used. The study was carried out in community pharmacies across the Asir region.

### Population and Setting

This study was conducted with community pharmacists practicing in the Asir region, which is in the southern province of Saudi Arabia. The total number of licensed community pharmacies in this region is 438.

### Sample Size and Sampling Procedure

The sample size was based on the number of community pharmacists in the Asir region (747) and determined by using a Raosoft sample size calculator (http://www.raosoft.com/samplesize.html) with a predetermined margin of error of 5% and a confidence level of 95%. In order to minimize erroneous findings and increase study reliability, the target sample size was set at 254 pharmacists. A non-probability convenience sampling method was used (16). Efforts were made to recruit the minimum sample size (254), however, only 211 pharmacists agreed to take part in the study.

### Study Period

The study was conducted between November 2020 and March 2021.

### Data Collection Form

The structured questionnaire was adapted from a previous study conducted in Turkey (17). The adaptation was conducted by altering the questions of the original survey to fit the context and aim of the current study. The final questions were then re-distributed into the study domains i.e., demographics and background, knowledge, attitude, and practice. The mapping of the original study questionnaire with this study is available in Appendix 1.

The data collection tool was piloted with 4 community pharmacists who met the study inclusion criteria. The online questionnaire was sent with an extra short answer question about their opinions and possible amendments to the questionnaire. Two questions, about whether selling non pharmaceuticals makes pharmacists less respected, and the other question was whether pharmacists had received formal education on non-pharmaceutical products in their undergraduate studies, were suggested and added. The data collection tool was finalized in the English language in the form of a self-administered web-based questionnaire. The findings of the pilot study were not included in the final results.

Face and content validation were conducted by two academics at the Clinical pharmacy department at Kin Khalid University who have expertise in the field of community pharmacy research. The questionnaire is available in Appendix 2.

The questionnaire was distributed by email to two of the largest pharmacy chains in the region.

### Inclusion Criteria

Being a licensed pharmacist in Saudi Arabia, working in a community pharmacy in the Asir region, having a minimum of Bachelor's degree certification or higher degree, and being willing to participate in the study.

### Exclusion Criteria

Pharmacy personnel working in other sectors including hospitals, industry, academia, and pharmacy regulation, and community pharmacists who are based outside the Asir region.

### Statistical Analysis

The collected data were cleared, entered and analyzed by using the Statistical Package for Social Sciences (SPSS) version 26.0 for the Mac. Demographic and background information were described in terms of frequencies. Knowledge (5 items) and attitude (5 items) used a Likert scale ranging from 1 (strongly disagree) to 5 (strongly agree). Practice (4 items) used a Likert scale ranging from 1 (never) to 5 (always). Scales for knowledge, attitude, and practice were categorized into themes. The distribution of the scale was presented in percentages, and using mean and SD. The internal consistency and reliability of the scales was assessed using Cronbach's alpha coefficient, with the minimum recommended level being 0.70.

### Ethics Consideration

An ethical clearance was given by The Ethical Committee of the Scientific Research, King Khalid University **(ECM#2020-169)—(HAPO**-06-B-001**)**. A written informed consent was collected from participants.

## Results

Demographics and background are summarized in Table 1. A total of 211 community pharmacists participated in the study. The majority of participants were male (72%) of a younger age between 21 and 30 (69.7%). Just over two thirds had a Bachelor's degree in pharmacy (71.1%) and around one third had a Pharm D degree, while a couple had a postgraduate degree in pharmacy. Just under two thirds (59.7%) of participants were recent graduates with work experience of <5 years.

**Table 1:** Demographics and background information.

**Demographics** ***n*** **=** **211**		
	**n**	**Percentage %**
**Gender**		
Male	152	72
Female	59	28
**Age**		
21–30	147	69.7
31–40	47	22.3
41–50	15	7.1
51–60	1	0.5
>60	1	0.5
**Education**		
Bachelor's degree in pharmacy	150	71.1
Doctor of Pharmacy (Pharm D)	59	28
Postgraduate degree in pharmacy	2	0.9
**Work experience in years**		
1–5	126	59.7
>5	85	40.3
**Background**		
Pharmacy location		
In local neighborhood	106	50.2
In a shopping mall or a main street	43	20.4
Attached to a hospital or healthcare centre	30	14.2
**14.2**	32	15.2
**Bestselling products in community pharmacy**		
Pharmaceutical products including prescribed medicines/ OTC	147	69.7
Non-pharmaceutical products	64	30.3

Half of the pharmacies included in the study (50.2%) were located in local neighborhoods, 20.4% were located in a main street, 15.2% were located in remote areas and 14.2% were attached to a healthcare facility. Products sold in community pharmacies were mainly pharmaceutical products (69.7%) compared to 30.3% nonpharmaceutical products.

**Figure 1:**
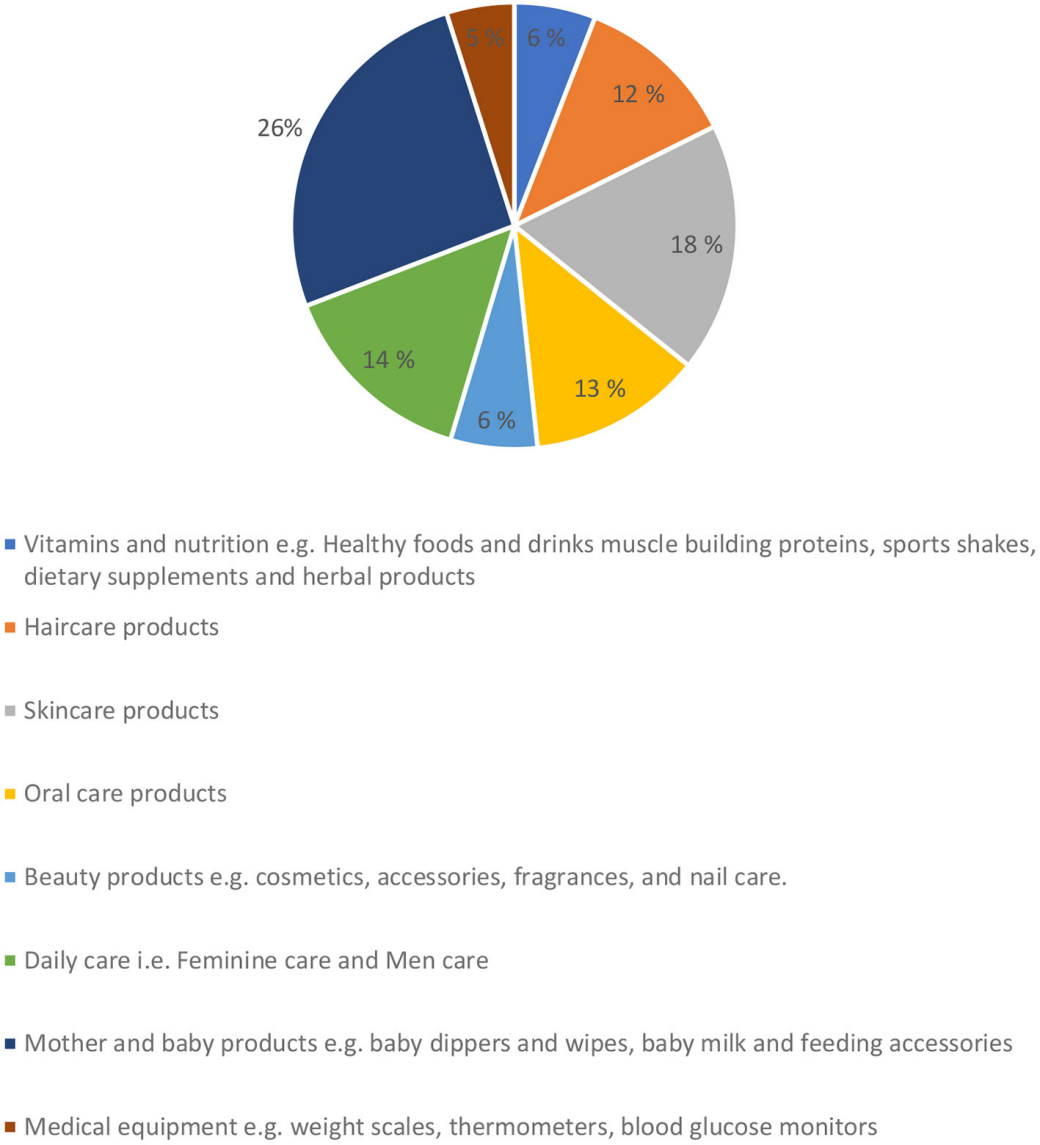
Bestselling non-pharmaceutical products sold in community pharmacy.

The most commonly sold non-pharmaceutical products were mother and baby products (27%) and skin care products (19%), whereas the least popular products were medical equipment (5%) and vitamins and nutrition (6%) (Figure 1).

The distribution of scale scores of the full set of 5 items of community pharmacists' knowledge toward non-pharmaceutical products is presented in Table 2. Responses ranged from 1 (strongly disagree) to 5 (strongly agree). All items were skewed toward 5 (strongly agree), indicating that pharmacists had good knowledge.

**Table 2:** Distribution of community pharmacists' prior knowledge and training toward nonpharmaceutical products from 1 (strongly disagree) to 5 (strongly agree), *n* = 211.

**Knowledge description**	**Distribution of responses (%)**					**Mean**	**SD**	**Skewness**
	**1**	**2**	**3**	**4**	**5**			
I was taught about non-pharmaceutical products during my undergraduate studies	7.6	**25.1**	13.3	35.5	18.5	3.32	1.246	−0.289
I received training courses about non-pharmaceutical products at my current pharmacy/job	4.3	9	11.4	40.3	35.1	3.92	1.09	−1.051
I self-studied non-pharmaceutical products	1.9	0.9	13.3	50.2	33.6	4.128	0.815	−1.198
I am keeping my knowledge up to date regarding non-pharmaceutical products	0.5	1.4	12.3	45	40.8	4.24	−0.758	−0.899
I am competent to provide information and advice on non-pharmaceutical care products	0.5	2.8	11.8	46.4	38.4	4.194	0.789	−0.945

The distribution of scale scores of the full set of 5 items of community pharmacists' attitudes toward nonpharmaceutical products presented in Table 3. Responses ranged from 1 (strongly disagree) to 5 (strongly agree). All items were skewed toward 5 (strongly agree), indicating that pharmacists have positive attitudes, i.e., marketing and providing information, keeping up to date.

**Table 3:** Distribution of community pharmacists' attitude toward non-pharmaceutical products from 1 (strongly disagree) to 5 (strongly agree), *n* = 211.

**Attitude description**	**Distribution of responses (%)**					**Mean**	**SD**	**Skewness**
	**1**	**2**	**3**	**4**	**5**			
1. Marketing and providing information about non-pharmaceutical products is a pharmacist's professional responsibility.	1.4	9.5	10.9	37	41.2	4.07	1.01	−1.02
2. Keeping updated on non-pharmaceutical products should be mandatory	1.4	5.7	18.5	39.8	35.5	4.01	0.948	−0.84
3. Pharmacists should pay more attention to non-pharmaceutical products as they are more profitable	2.4	4.7	23.2	37.9	31.8	3.919	0.975	−0.771
4. Marketing/promoting certain non-pharmaceutical products is an essential duty for pharmacists	5.2	7.6	22.3	37	28	3.748	1.1	−0.776
5. Selling non pharmaceuticals makes pharmacists less respected	7.6	24.6	25.6	20.9	21.3	3.23	1.25	−0.03

The distribution of scale scores of the full set of 4 items of community pharmacists' practice toward non-pharmaceutical products presented in Table 4. Responses ranged from 1 (never) to 5 (always). All items were skewed toward 5 (always), indicating that pharmacists have good practice.

**Table 4:** Distribution of community pharmacists' practice toward non-pharmaceutical products from 1 (never) to 5 (always), *n* = 211.

**Practice description**	**Distribution of responses (%)**					**Mean**	**SD**	**Skewness**
	**1**	**2**	**3**	**4**	**5**			
Are you involved in promoting/marketing non-pharmaceutical products?	19.9	21.3	31.3	22.3	5.2	2.7	1.16	−0.011
Do you advise your clients about the use of certain non-pharmaceutical products?	11.4	10.9	18	33.2	26.5	3.5	1.29	−0.63
Do you get inquiries related to non-pharmaceutical products?	8.1	8.5	18	34.6	30.8	3.7	1.21	−0.816
Do you sell non-pharmaceutical products	14.2	10.9	25.6	26.1	23.2	3.3	1.32	−0.395

Table 5 presents the distribution of the variables being investigated. These scales are treated as continuous variables, ranging from 1 (strongly disagree) to 5 (strongly agree) for knowledge and attitude, and 1 (never) to 5 (always) for practice. The mean value of the overall scales— knowledge, attitude, and practice— were 3.96, 3.79 and 3.3, respectively. All scales had a Cronbach alpha coefficient >0.7, indicating inter-item reliability.

**Table 5:** Distribution and internal consistency of knowledge, attitude, and practice of community pharmacists' toward non-pharmaceutical products.

**Description of scale**	**Distribution of responses (%)**					**Mean**	**SD**	**Skew**	**Cronbach alpha**
	**<1**	**<2**	**<3**	**<4**	**<5**				
Knowledge (Items 1,2,3,4,5)	0	0.9	11.8	57.8	18.5	100	0.686	−0.686	0.759
Attitude (Items 1,2,3,4,5)	0	1.4	18.5	67.8	35.1	100	0.72	−0.33	0.705
Practice (Items 1,2,3,4)	5.2	15.6	37.4	79.6	33.6	100	1.01	−0.625	0.825

## Discussion

This study was conducted to assess community pharmacists' knowledge, attitude and practice toward non-pharmaceutical products. Previous national studies conducted in community settings focused on pharmacy practice related to prescribed and OTC medications such as pharmaceutical care, adverse drug reaction reporting, dispensing antibiotics without prescriptions, counseling skills, medication safety during pregnancy, mental health illness, diabetes, asthma, inhaler use, cardiac conditions, herbal medications, and specific drugs such as isotretinoin (18, 19).

The government launched an initiative to encourage public-private partnerships for effective delivery of preventative primary health care in Vision 2030 (2). The Saudi Ministry of Health introduced an initiative to enhance health services named “Wasfaty”. It permits beneficiaries of government health services to access medications form community pharmacies (private sector) located in their neighborhood instead of obtaining them free of charge from pharmacies located inside hospitals and primary healthcare centers (20). As a result, public exposure to non-pharmaceutical products has increased when visiting community pharmacies to dispense their prescriptions.

The study revealed that although community pharmacies sell non-pharmaceutical products, these are sold in lower proportions than pharmaceutical products (69.7%). The most popular non-pharmaceutical products were mother and baby products (26%) and skin care products (19%), whereas the least popular products were medical equipment (5%), and vitamins and nutrition (6%).

Similarly, a previous study conducted in Turkey found that the most common non-pharmaceutical products are food supplements (87.3%), and mother and baby products (86.5%), while the least common were sports products and foods (5.7%) (17).

The data suggest that community pharmacists were well-equipped with knowledge about non-pharmaceutical products as they were educated on this topic during their undergraduate studies. Moreover, participants of the study stated that they agreed or strongly agreed with keeping up to date regarding their area of practice through self-education. In 2020, the total number of community pharmacists in Saudi Arabia was 17,815 of which only 2,271 were Saudi (21) It is worth mentioning that some participants are non-Saudi, so they come from different education and training backgrounds. However, Saudi participants are King Khalid University graduates as this has the only pharmacy college in the region.

More attention has been given to pharmacy practice in the community setting by the education body, King Khalid University (KKU) (22, 23). Need-based education to serve local communities was driven by Saudi Vision 2030 in terms of focusing on primary and preventative care as well as strengthening the private-government partnership in providing healthcare. Some undergraduate courses such as Professional Pharmacy Practice Lab I, non-prescription drugs and Introductory Pharmacy Practice Experiences (IPPEs), which are part of the didactic curriculum, aim to prepare students to practice in the community pharmacy sector. Advanced Pharmacy Practice Experiences (APPEs) in community pharmacies provide students with real life experiences of working in this setting (4). Recent employment rules and regulations were issued to enhance the participation of Saudi pharmacists in the community pharmacy sector in two the stages: 20 % by 2020 and 30 % by 2021 (3).

Early career training is also offered by one of the biggest pharmacy chains in the country with more than 800 branches in 145 cities and villages; new pharmacy graduates are offered a 3-month intensive training course before entering the job market. Another large chain of community pharmacy with more than 800 branches in over 100 cities also offers training opportunities for newly-hired pharmacists. This was clearly stated by the majority of participants who agreed or strongly agreed that they received training to work in this sector (24).

Pharmacists showed positive attitudes toward non-pharmaceutical products in terms of understanding what employers expect from them. The majority of pharmacists believed that marketing, making financial profits, providing advice and counseling for customers, and keeping up to date with advancements in the field are the core rules for employees in this area of practice. Reviewing job requirements by the biggest pharmacy chains in the country showed that pharmacists are expected to manage the premises, process prescriptions, provide counseling on medication and education on medical devices, monitor key sales performance targets, process payments and reconciliations by handling the cash register, monitor operating staff, ensure compliance with health and safety policies and procedures and attend continuous professional development activities and workshops. Hence, pharmacists were fulling the professional obligations by providing counseling while ensuring the survival of the business by making financial profits by selling non-pharmaceutical products.

Similar findings were reported in a previous study in Turkey where participants believed that non-pharmaceutical products should only be sold in community pharmacies to help with revenue and profits. They also believed that providing counseling for customers on these products is the duty of the pharmacist (17).

The study findings also indicated that pharmacists had positive practice toward non-pharmaceutical products, i.e., marketing promoting products, providing advice and answering inquires, as well as selling these products. Hence, there was no knowledge-attitude-practice gap among community pharmacists in the Asir Region.

The study has some limitations, including the self-evaluation of pharmacists' knowledge, in particular the fact that their views on continuous professional development about non-pharmaceutical products could be biased. The majority of participants were male, as the law to easing the restrictions on female pharmacists to work in community settings has been newly implemented. Data collection was limited to a single point in time, so changes over time were not assessed.

## Conclusion

The study revealed that the most commonly sold non-pharmaceutical products were mother and baby products and skin care products. Community pharmacists showed considerable knowledge pertaining to non-pharmaceutical products i.e. they have been educated and trained well during their undergraduate studies, they also self-educated themselves regarding nonpharmaceutical products. Respondents demonstrated positive attitudes toward the non-pharmaceutical products and were interested in expanding their knowledge on the topic through continuing education and believed that marketing and promoting were fundamental duty for pharmacists. Additionally, they showed social accountability by assuming responsibility for providing patient counseling on non-pharmaceutical products. Pharmacists were involved in selling, marketing, providing advice and answering inquiries related to non-pharmaceutical products.

## Data Availability Statement

The raw data supporting the conclusions of this article will be made available by the authors, without undue reservation.

## Ethics Statement

The studies involving human participants were reviewed and approved by the Ethical Committee of the Scientific Research, King Khalid University. The patients/participants provided their written informed consent to participate in this study.

## Author Contributions

The author confirms being the sole contributor of this work and has approved it for publication.

## Conflict of Interest

The reviewer AA declared a shared affiliation with the author DA, to the handling editor at the time of review. The author declares that the research was conducted in the absence of any commercial or financial relationships that could be construed as a potential conflict of interest.

## Publisher's Note

All claims expressed in this article are solely those of the authors and do not necessarily represent those of their affiliated organizations, or those of the publisher, the editors and the reviewers. Any product that may be evaluated in this article, or claim that may be made by its manufacturer, is not guaranteed or endorsed by the publisher.

## Abbreviations

SCFHS: The Saudi Commission for Health Specialties
SPLE: Saudi Pharmacists Licensure Examination
OTC: Over-the counter
SFDA: The Saudi Food and Drug Administration
MOH: Ministry of Health
Pharm D: Doctor of Pharmacy
KKU: King Khalid University
CME: Continuing Medical Education.
