# Supplementary material for: Knowledge, Attitude and Practice of Community Pharmacists Toward Non-pharmaceutical Products in Saudi Arabia
Source: Front Public Health. 2022 Apr 29;10:771308. doi: 10.3389/fpubh.2022.771308 (PMC9099022; doi:10.3389/fpubh.2022.771308)
Supplement: Supplementary file 1 [file Table_1.docx]

**Appendix 1 Mapping of data collection tool questions**

| **The original survey developed by Gulpinar et al (2019)** | **The adapted survey used in the present study** |
| --- | --- |
| Table 1  Location of community pharmacies participated in the survey | Table 1  Pharmacy location |
| Table 2.  The sales percentages of NPMPs held by pharmacists participated in the survey | Table 1  Type of products sold in community pharmacy |
| - | Table 1  Pharmacists demographics  Gender, age, education. |
| **Table 3.**  **Opinions of community pharmacists on possible legal regulations about NPMPs.** |  |
| NPMPs should be sold only in pharmacies |  |
| I think public should ask for information concerning NPMPs from pharmacists. | **Table 4. Do you get inquiries related to non-pharmaceutical products?** |
| The public should be informed by direct-to-consumer advertising about NPMPs |  |
| I think we need to go through the data matrix applications in NPMPs. |  |
| Some weight control products, some food supplements and dermocosmetic products should be added to Social Security reimbursement list |  |
| I believe that NPMPs direct-to consumer advertising can inform the patient adequately | **Table 3. Marketing and providing information about non-pharmaceutical products is a pharmacist’s professional responsibility.** |
| I believe that direct-to-consumer advertising can increase the sales of NPMPs | **Table 4. Do you sell non-pharmaceutical products** |
| I believe that direct-to-consumer advertising can increase the consumption of NPMPs | **Table 3. Marketing and providing information about non-pharmaceutical products is a pharmacist’s professional responsibility.** |
| **Table 4. Attitudes of community pharmacists about NPMPs that they have in their pharmacies.** |  |
| I much prefer NPMPs because it is more profitable than prescription medicines. | **Table 3. Pharmacists should pay more attention to non-pharmaceutical products as they are more profitable** |
| I definitely recommend nutritional supplements to my patients who want nonprescription drugs | **Table 4. Do you advise your clients about the use of certain non-pharmaceutical products?** |
| I do not give information about food supplements without a request from client. |  |
| I recommend more NPMP according to the needs of the client that has better economic income | **Table.3 Marketing/promoting certain non-pharmaceutical products is an essential duty for pharmacists** |
| **Table 5. Community pharmacists’ opinions about the regulations on NPMPs.** |  |
| With the sale of NPMPs, pharmacies remain standing | **Table 3. Pharmacists should pay more attention to non-pharmaceutical products as they are more profitable** |
| I think request information from pharmacist about NPMPs is more than medicines. | **Table 3. Do you get inquiries related to non-pharmaceutical products?**  **Table 1. I am competent to provide information and advice on non-pharmaceutical care products** |
| I think the spread of NPMPs increases sales rate from the internet |  |
| I think that continuous learning organized regarding NPMPs can be helpful for pharmacists. | **Table 3. Keeping updated on non-pharmaceutical products should be mandatory**  Table 2.  I self-studied non-pharmaceutical products  I am keeping my knowledge up to date regarding non-pharmaceutical products  I received training courses about non-pharmaceutical products at my current pharmacy/job |
|  | **Table.3 Selling non pharmaceuticals makes pharmacists less respected** |
|  | Table 2. I was taught about non-pharmaceutical products during my undergraduate studies |

**Appendix 2Data collection tool**

**Knowledge, attitude and practice of community pharmacists towards non-pharmaceutical products in Saudi Arabia**

Dear pharmacist,

You are invited to take part in a study assessing Knowledge, attitude and practice of community pharmacists towards non-pharmaceutical products in Saudi Arabia. An ethical clearance was obtained from King Khalid University ethics committee. I thank you in advance for helping the researcher in filling this survey. The survey takes about 5 minutes to fill and is completely voluntary. The information you share for this study will remain confidential. No identifying information will be disclosed in the reporting process. Thank you for your participation

**Please answer the following questions**

**By completing this survey, you provide your written informed consent to be a part of this study**

1. Yes
2. No

**Demographics and background information**

**Age**

1. 21-30
2. 31-40
3. 41-50
4. 51-60
5. >60

**Gender**

1. Male
2. Female

**Education level**

1. First degree in pharmacy
2. Postgraduate degree in pharmacy

**Work experience in years**

1. 1-5 (fresh graduate)
2. >5

**Pharmacy location**

1. In local neighbourhood
2. In a shopping mall or a main street
3. Attached to a hospital or healthcare centre
4. In a remote area

**Bestselling products in community pharmacy**

1. Pharmaceutical products including prescribed medicines/ OTC
2. Non-pharmaceutical products

**What is the top-selling nonpharmaceutical products is in your pharmacy?**

1. Haircare products
2. Skincare products
3. Oral care products
4. Beauty products such as accessories, nail care products , fragrances, cosmetics
5. Daily care i.e. feminine care and men care
6. Medical equipment such as weight scales, thermometers, blood glucose monitor
7. Mother and baby products e.g. baby diapers and wipes, baby milk, and feeding accessories

Please rate the following statements regarding prior knowledge and training towards nonpharmaceutical products using a scale from strongly disagree to strongly agree

| Statement | **Strongly disagree** | **Disagree** | **Neutral** | **Agree** | **Strongly agree** |
| --- | --- | --- | --- | --- | --- |
| **I was taught about non-pharmaceutical products during my undergraduate studies** |  |  |  |  |  |
| **I received training courses about non-pharmaceutical products at my current pharmacy/job** |  |  |  |  |  |
| **I self-studied non-pharmaceutical products** |  |  |  |  |  |
| **I am keeping my knowledge up to date regarding non-pharmaceutical products** |  |  |  |  |  |
| **I am competent to provide information and advice on non-pharmaceutical care products** |  |  |  |  |  |

Please rate the following statements regarding **attitude towards non-pharmaceutical products**

| Statement | Strongly disagree | Disagree | Neutral | Agree | Strongly agree |
| --- | --- | --- | --- | --- | --- |
| **Marketing and providing information about non-pharmaceutical products is a pharmacist’s professional responsibility.** |  |  |  |  |  |
| **Keeping updated on non-pharmaceutical products should be mandatory** |  |  |  |  |  |
| **Pharmacists should pay more attention to non-pharmaceutical products as they are more profitable** |  |  |  |  |  |
| **Marketing/promoting certain non-pharmaceutical products is an essential duty for pharmacists** |  |  |  |  |  |
| **Selling non pharmaceuticals makes pharmacists less respected** |  |  |  |  |  |

Please rate the following statements regarding **practices towards non-pharmaceutical products** **using a scale from never to always**

| **Statement** | **Never** | **Rarely** | **Sometimes** | **Often** | **Always** |
| --- | --- | --- | --- | --- | --- |
| Are you involved in promoting/marketing non-pharmaceutical products? |  |  |  |  |  |
| Do you advise your clients about the use of certain non-pharmaceutical products? |  |  |  |  |  |
| Do you get inquiries related to non-pharmaceutical products? |  |  |  |  |  |
| Do you sell non-pharmaceutical products |  |  |  |  |  |
